# Inhibitory effect of toothbrush monofilament containing surface pre-reacted glass-ionomer (S-PRG) filler on *Streptococcus mutans*

**DOI:** 10.1038/s41598-020-80646-x

**Published:** 2021-01-08

**Authors:** Saaya Matayoshi, Ryota Nomura, Takahiro Kitamura, Rena Okawa, Kazuhiko Nakano

**Affiliations:** Division of Oral Infections and Disease Control, Department of Pediatric Dentistry, Osaka University Graduate School of Dentistry, 1-8 Yamada-oka, Suita, Osaka 565-0871 Japan

**Keywords:** Microbiology, Bacteria, Biofilms

## Abstract

The oral environment affects not only oral health, but also general health, and the importance of oral self-care has recently been recognised. Although toothbrushes are the most important self-care product, there are few toothbrushes that have an inhibitory effect on oral bacteria. In the present study, monofilaments used for toothbrushes containing surface pre-reacted glass-ionomer (S-PRG) filler (a component recently applied to various dental materials) were developed. Among nylon and polyester monofilaments commonly used for toothbrushes, nylon monofilaments can accommodate more S-PRG filler than polyester monofilaments, resulting in greater release of ions from the S-PRG filler. These monofilaments containing S-PRG filler formed less biofilm containing *Streptococcus mutans*, a major pathogen of dental caries, than monofilaments without S-PRG filler. Moreover, *S. mutans* adhering to monofilaments containing S-PRG filler were more easily exfoliated and eliminated than those adhering to monofilaments without S-PRG filler. Such inhibitory effects on *S. mutans* were more marked in nylon monofilaments than in polyester monofilaments. These findings that monofilaments containing S-PRG filler can release ions and have an inhibitory effect on *S. mutans* suggest that they may be an effective material for toothbrushes.

## Introduction

Oral health has recently been recognised as being closely associated with general health and quality of life^1^. To maintain oral health, regular check-ups and professional tooth cleaning by dentists or dental hygienists (professional care) as well as daily prevention at home (self-care) are of importance^2,3^. Most oral self-care products such as toothpastes and mouthwashes contain various components with an inhibitory effect on oral bacteria^4,5^.

Daily self-care using a toothbrush is one of the most important methods of reducing oral bacterial colonisation^6,7^. After brushing, many oral bacteria adhere to the bristles of the toothbrush^8^. *Streptococcus mutans*, a major causative agent of dental caries^9^, can survive on the bristles for more than 8 h after brushing^10,11^. Although the presence of *S. mutans* on the toothbrush sometimes interferes with oral health^12^, there are no studies focusing on toothbrushes that have an inhibitory effect on *S. mutans*.

Surface pre-reacted glass-ionomer (S-PRG) filler is a bioactive functional glass that releases six ions: borate (BO_3_^3−^), aluminium (Al^3+^), silicate (SiO_3_^2−^), strontium (Sr^2+^), sodium (Na^+^) and fluoride (F^−^)^13^. S-PRG filler has been used in various dental materials such as composite resins, cements and bonding agents^14,15^. S-PRG filler has some beneficial functions for maintaining oral health, such as anti-plaque formation, acid neutralisation, and enamel remineralisation^16–18^.

S-PRG filler can inhibit bacterial growth and biofilm formation of *S. mutans*, and parts of these mechanisms have been reported in our previous study^19^. Recently, S-PRG filler has been added to some dental materials, such as tooth surface coating materials and resin sealants, for caries prevention^18,20^. Thus, although S-PRG filler has been added to various professional care dental materials, it has not yet been added to self-care products.

In the present study, we developed a toothbrush monofilament containing S-PRG filler as a novel self-care product with anti-cariogenic properties. We analysed the properties of the monofilaments required for anti-cariogenic properties such as ion release activity and tensile strength. The inhibitory effect of monofilaments containing S-PRG filler on *S. mutans* was also analysed.

## Results

### Analysis of the internal structure of monofilaments containing S-PRG filler

**Figure 1:**
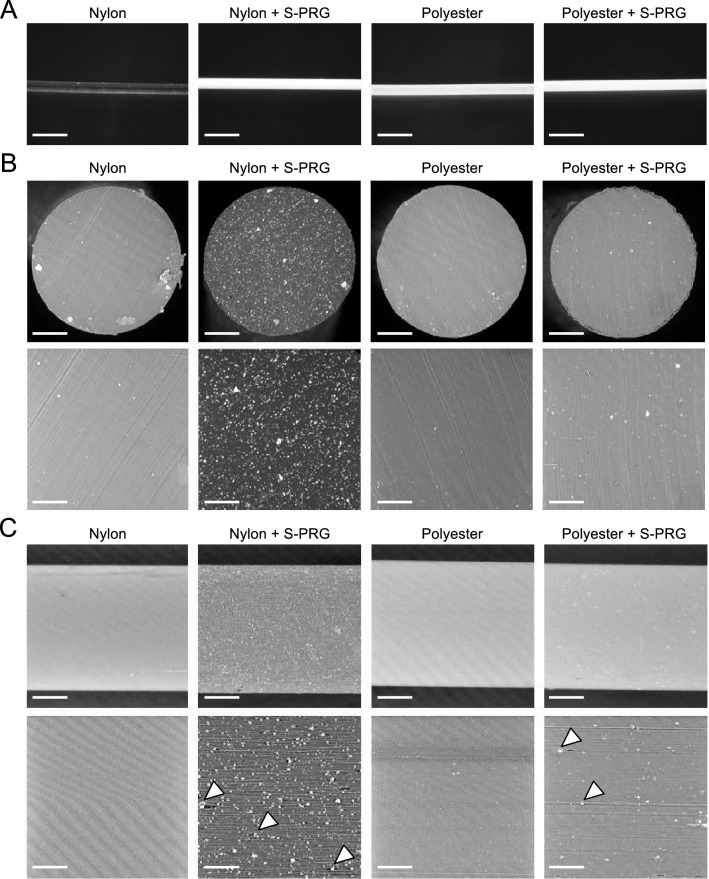
Structure of monofilaments with or without S-PRG filler. (**A**) Representative images of monofilaments (bar = 600 μm). (**B**) Transverse sections of monofilaments in scanning electron microscope (SEM) images. Upper panels show low-magnification images (bar = 50 μm) and lower panels show high-magnification images (bar = 30 μm). White particulate materials in the nylon and polyester monofilaments containing S-PRG filler are S-PRG filler. (**C**) Side structure of monofilaments in SEM images. Upper panels show low-magnification images (bar = 50 μm) and lower panels show high-magnification images (bar = 20 μm). Arrowheads indicate S-PRG filler that has penetrated into the monofilament fibres.

Two types of monofilaments for toothbrushes with a diameter of 200 μm were manufactured using nylon and polyester (Fig. 1A). In the manufacturing process, monofilaments containing S-PRG filler were also prepared. To spin a uniform monofilament, it was possible to contain up to 20% of S-PRG filler in the nylon monofilaments and 1.4% of S-PRG filler in the polyester, respectively. Therefore, these concentrations of S-PRG filler were used in the monofilaments in the following experiments. The clear nylon monofilaments changed to a white colour with the addition of S-PRG filler, while no colour change was observed in the white polyester monofilaments with or without S-PRG filler. To confirm the presence of S-PRG filler in the monofilaments, the transverse section and side structure of the monofilaments were observed using a scanning electron microscope (SEM) (Fig. 1B). Monofilaments containing S-PRG filler were studded almost uniformly with particles that were tens of nanometres to several micrometres in diameter. The nylon monofilaments had more particles than the polyester monofilaments. In the monofilaments without S-PRG filler, no particles were observed in either the nylon or the polyester monofilaments. Enlarged images of the side view of the monofilaments revealed that the S-PRG filler penetrated into the monofilament fibres (Fig. 1C). The particles included some elements corresponding to ions released from the S-PRG filler; this was confirmed by energy dispersive X-ray spectroscopy analysis (Supplementary Tables 1, 2).

### Ion concentration released from monofilaments containing S-PRG filler

**Figure 2:**
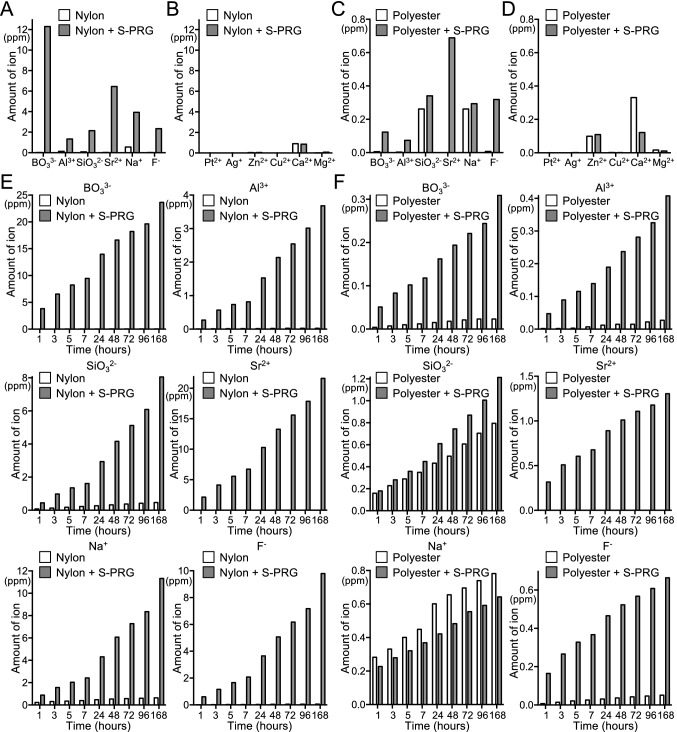
Ion concentrations released from monofilaments. (**A**–**D**) Ion concentrations released from monofilaments into sterilised distilled water after mixing for 18 h. Amounts of ions derived from S-PRG filler (**A**) and not derived from S-PRG filler (**B**) in nylon monofilaments. Amounts of ions derived from S-PRG filler (**C**) and not derived from S-PRG filler (**D**) in polyester monofilaments. (**E**, **F**) Cumulative ion concentrations released from monofilaments into sterilised distilled water, which was replaced after 1, 3, 5, 7, 24, 48, 72, 96, 168 h. Nylon monofilaments (**E**) and polyester monofilaments (**F**).

The amounts of six ions (BO_3_^3−^, Al^3+^, SiO_3_^2−^, Sr^2+^, Na^+^ and F^−^) released from the S-PRG filler contained in the monofilaments were measured. First, the monofilaments were immersed in sterilised distilled water and mixed for 18 h, and the amount of the ions released into the water was measured. More than 1 ppm of all ions derived from S-PRG were released from the nylon monofilaments containing S-PRG filler (Fig. 2A), with BO_3_^3−^ released in the highest concentration. No such ion release was observed from the nylon monofilaments without S-PRG filler. In contrast, less than 1 ppm of the ions platinum (Pt^2+^), silver (Ag^+^), zinc (Zn^2+^), copper (Cu^2+^), calcium (Ca^2+^) and magnesium (Mg^2+^), none of which are derived from S-PRG filler, was released from any of the nylon monofilaments (Fig. 2B). Polyester monofilaments containing S-PRG filler released lower levels of the S-PRG-derived ions than did the nylon monofilaments containing S-PRG filler (Fig. 2C), and most of the ions not derived from S-PRG filler were released at lower levels than those derived from S-PRG filler (Fig. 2D).

The sterilised distilled water in which the monofilaments were immersed was replaced 1, 3, 5, 7, 24, 48, 72, 96 and 168 to analyse whether the ion release continued or not. In the nylon monofilaments containing S-PRG filler, all ions were continuously released even after 168 h (1 week) of immersion in sterile distilled water (Fig. 2E). The release of ions from polyester monofilaments containing S-PRG filler was less than that of nylon monofilaments containing S-PRG filler (Fig. 2F).

### Tension test using monofilaments containing S-PRG filler

**Figure 3:**
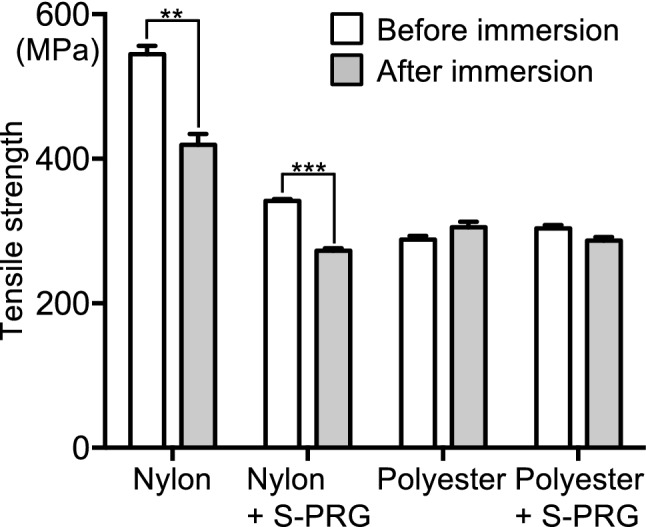
Tensile strength of monofilaments before and after immersion in water. Significant differences were determined using a chi-square test. **P < 0.01, ***P < 0.001 between the groups.

To analyse the effect of S-PRG filler on the mechanical strength of monofilaments, a tension test using monofilaments with or without immersion in water was performed (Fig. 3). Nylon monofilaments recorded higher tensile strength than polyester monofilaments both with and without S-PRG filler, although the tensile strength of the nylon monofilaments decreased by approximately 35% in the presence of S-PRG filler. Regardless of the presence or absence of S-PRG filler, the tensile strength of the nylon monofilaments was reduced by approximately 20% with immersion in sterilised distilled water compared with no immersion. In contrast, the tensile strength of polyester monofilaments with or without S-PRG filler was stable and unaffected by immersion in water. Although the tensile strength of the nylon monofilaments decreased with the addition of S-PRG filler and immersion in water, all nylon monofilaments had a tensile strength equal to or greater than that of polyester monofilaments without S-PRG filler.

### Inhibitory effects of monofilaments containing S-PRG filler on biofilm formation of *S. mutans*

**Figure 4:**
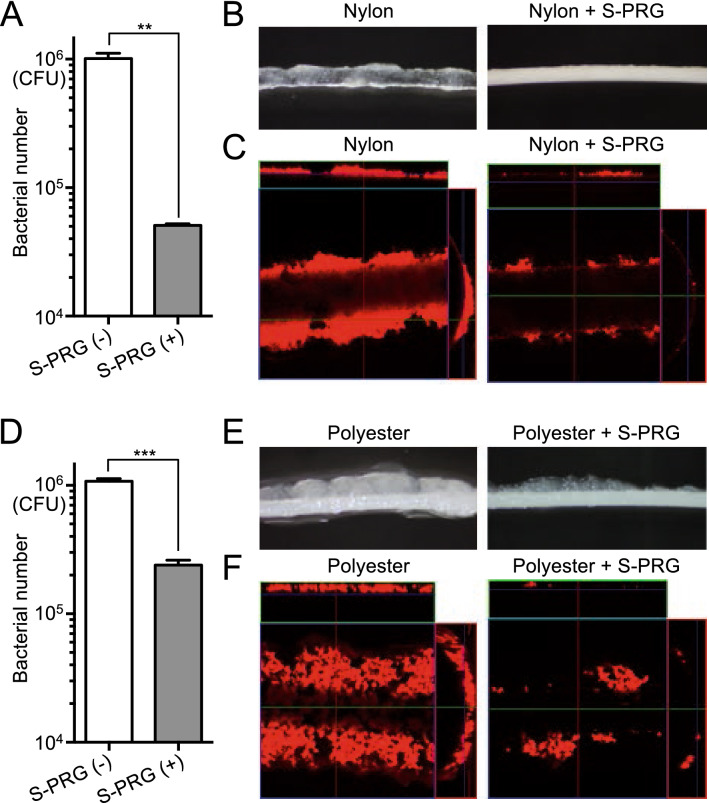
Biofilm formation of *S. mutans* on monofilaments. (**A**) Number of *S. mutans* adhering to nylon monofilaments with or without S-PRG filler. Significant differences were determined using a chi-square test. **P < 0.01 between the groups. (**B**) Representative stereomicroscopic images of nylon monofilaments with and without S-PRG filler. (**C**) Representative confocal laser microscopic images of nylon monofilaments with and without S-PRG filler. Orthogonal views of z stacks composed of *S. mutans* cultured on nylon monofilaments. Bacterial cells adhering to the nylon monofilaments are stained red. (**D**) Number of *S. mutans* adhering to polyester monofilaments with and without S-PRG filler. Significant differences were determined using a chi-square test. ***P < 0.001 between the groups. (**E**) Representative stereomicroscopic images of polyester monofilaments with and without S-PRG filler. (**F**) Representative confocal laser microscopic images of polyester monofilaments with and without S-PRG filler. Orthogonal views of z stacks composed of *S. mutans* cultured on polyester monofilaments. Bacterial cells adhering to the polyester monofilaments are stained red.

To examine the amount of *S. mutans* adhering to the monofilaments, a monofilament and *S. mutans* strain MT8148 were added to a 1% sucrose solution containing Brain Heart Infusion (BHI; Difco Laboratories, Detroit, MI, USA) broth and incubated at 37 °C for 18 h to form a biofilm on the monofilament. Approximately 1 × 10^6^ CFU of *S. mutans* adhered to the nylon monofilament without S-PRG filler, whereas the amount of *S. mutans* adhering to the nylon monofilament containing S-PRG filler decreased to approximately 1/20 (Fig. 4A). Stereomicroscopic and confocal laser microscopic findings showed that the nylon monofilaments without S-PRG filler formed a high density biofilm (Fig. 4B, C), while the density of the biofilm on the nylon monofilaments containing S-PRG filler was drastically reduced. Additionally, approximately 1 × 10^6^ CFU of *S. mutans* adhered to the polyester monofilaments without S-PRG filler, whereas the amount of *S. mutans* adhering to the polyester monofilaments containing S-PRG filler was decreased to about 1/5 (Fig. 4D). These results were confirmed in the stereoscopic and confocal laser microscopic findings (Fig. 4E, F).

### Inhibitory effects of monofilaments containing S-PRG filler on the adhesive properties of *S. mutans*

**Figure 5:**
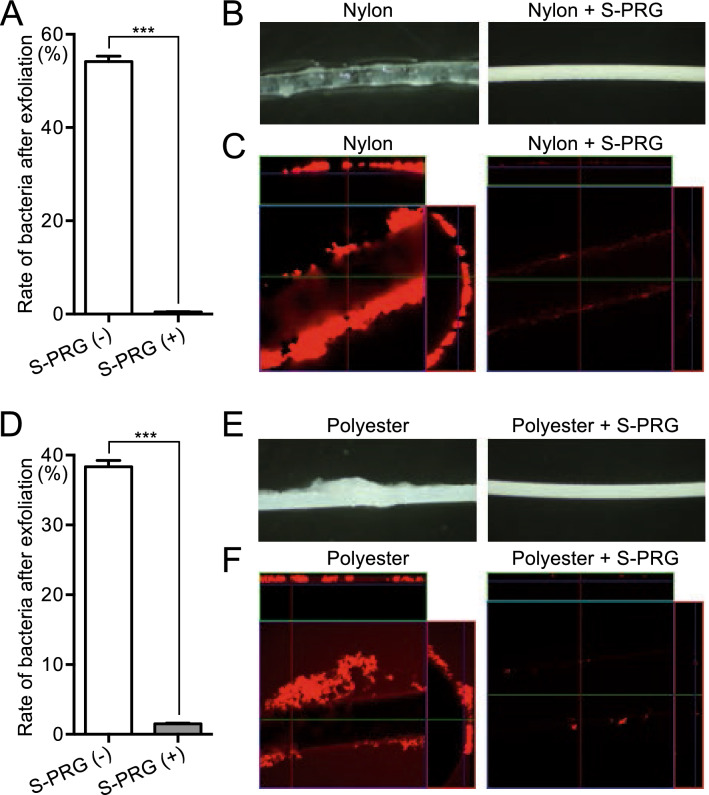
Adhesive properties of *S. mutans* to monofilaments. (**A**) Rate of *S. mutans* remaining on nylon monofilaments with and without S-PRG filler after vortexing for 3 s. Significant differences were determined using a chi-square test. ***P < 0.001 between the groups. (**B**) Representative stereomicroscopic images of nylon monofilaments with and without S-PRG filler. (**C**) Representative confocal laser microscopic images of nylon monofilaments with and without S-PRG filler. Orthogonal views of z stacks composed of *S. mutans* adhering to nylon monofilaments. Bacterial cells adhering to the nylon monofilaments are stained red. (**D**) Rate of *S. mutans* remaining on polyester monofilaments with and without S-PRG filler after vortexing for 3 s. Significant differences were determined using a chi-square test. ***P < 0.001 between the groups. (**E**) Representative stereomicroscopic images of polyester monofilaments with and without S-PRG filler. (**F**) Representative confocal laser microscopic images of polyester monofilaments with and without S-PRG filler. Orthogonal views of z stacks composed of *S. mutans* adhering to polyester monofilaments. Bacterial cells adhering to the polyester monofilaments are stained red.

To measure the strength of the adhesive properties of *S. mutans* to monofilaments, *S. mutans* biofilm was grown on each monofilament as described above. The monofilament was vortexed for 3 s to separate *S. mutans* from the monofilament. Thereafter, the amount of *S. mutans* remaining on the monofilament was measured by determining the number of bacteria detached and the number remaining on the monofilament. More than 50% of *S. mutans* remained on the nylon monofilaments without S-PRG filler (Fig. 5A–C), whereas most *S. mutans* were separated from the nylon monofilaments containing S-PRG filler, which was a significant difference (*P* < 0.001). The numbers of bacteria detached and remaining on nylon monofilaments with and without S-PRG filler are shown in Supplementary Fig. 1A, B. Polyester monofilaments without S-PRG filler retained approximately 40% of *S. mutans* after vortexing (Fig. 5D–F), whereas less than 2% of *S. mutans* remained on polyester monofilaments containing S-PRG filler, which was a significant difference (*P* < 0.001). The numbers of bacteria detached and remaining on polyester monofilaments with or without S-PRG filler are shown in Supplementary Figs. 1C and 1D.

### Chronological changes in the number of *S. mutans* on S-PRG filler-containing monofilaments

**Figure 6:**
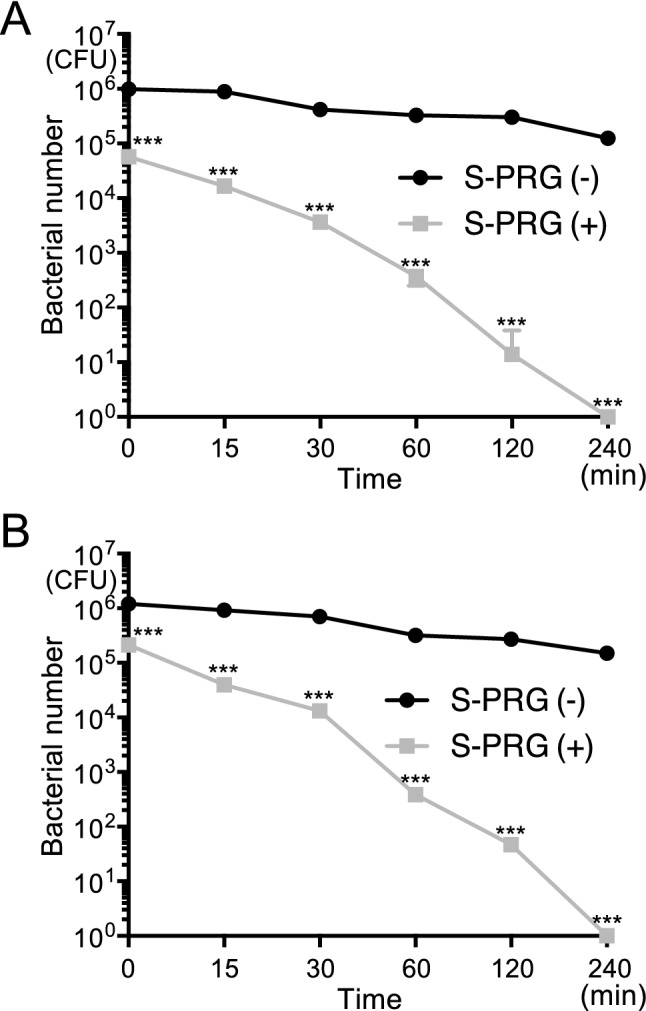
Chronological changes in the number of *S. mutans* on monofilaments with S-PRG filler. (**A**) Number of *S. mutans* adhering to nylon monofilaments with and without S-PRG filler at each time point. Significant differences were determined using a chi-square test. ***P < 0.001 compared with nylon monofilaments without S-PRG filler at each time point. (**B**) Number of *S. mutans* adhering to polyester monofilaments with and without S-PRG filler at each time point. Significant differences were determined using a chi-square test. ***P < 0.001 compared with polyester monofilaments without S-PRG filler at each time point.

After *S. mutans* biofilm was grown on the monofilaments as described above, the monofilaments were removed from BHI broth containing 1% sucrose and chronological changes in the number of *S. mutans* present on the monofilaments under dry conditions were analysed. Nylon monofilaments without S-PRG filler showed a moderate decrease in bacterial numbers, whereas nylon monofilaments containing S-PRG filler had almost no bacteria after 240 min (Fig. 6A). A moderate decrease in bacterial numbers was observed on polyester monofilaments without S-PRG filler, while a drastic decrease was observed on polyester monofilaments with S-PRG filler (Fig. 6B).

### Inhibitory effects of monofilaments containing S-PRG filler on bacterial growth of *S. mutans*

**Figure 7:**
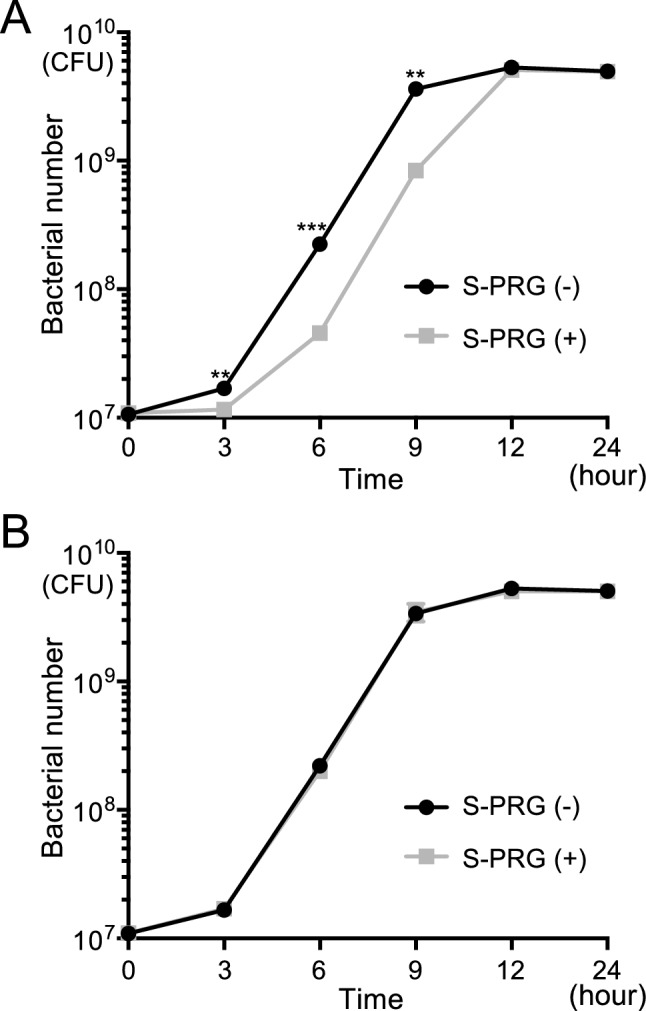
Bacterial growth of *S. mutans* in the presence of monofilaments. (**A**) Bacterial growth of *S. mutans* in the presence of nylon monofilaments with and without S-PRG filler at each time point. Significant differences were determined using a chi-square test. **P < 0.01, ***P < 0.001 compared with nylon monofilaments without S-PRG filler at each time point. (**B**) Bacterial growth of *S. mutans* in the presence of polyester monofilaments with and without S-PRG filler at each time point.

To investigate the effect of monofilaments on bacterial growth, *S. mutans* was cultured in BHI broth in the presence of 100 pieces of each monofilament. Nylon monofilaments containing S-PRG filler inhibited bacterial growth significantly more than nylon monofilaments without S-PRG filler at 3, 6 and 9 h after the start of the experiment (*P* < 0.01) (Fig. 7A). In contrast, there was no difference in the number of *S. mutans* between polyester monofilaments with or without S-PRG filler at any time point (Fig. 7B).

## Discussion

Daily self-care products such as toothpastes and mouthwashes containing antibacterial agents have been developed^4,5^. However, toothbrushes, which are considered to be the most important tool in self-care, have rarely been developed to exert an inhibitory effect on bacteria^21^. In the present study, we developed monofilaments containing S-PRG filler, which is a novel dental material with an inhibitory effect on *S. mutans*.

S-PRG filler is a bioactive functional glass that enables the release of six types of ions (BO_3_^3−^, Al^3+^, SiO_3_^2−^, Sr^2+^, Na^+^ and F^−^) by forming an equilibrium state with the external environment^13^. S-PRG fillers are known to have oral antibacterial properties, as well as their properties of strengthening teeth and neutralising acid^16–18^. Recently, S-PRG filler has been applied to various dental materials such as composite resins, coating materials, and resin sealants^14,15,18^. S-PRG fillers aggregates range in size from tens of nanometres to several micrometres (average 4.1 μm) in diameter^22^, and can be added all over the monofilaments as observed under an electron microscope.

Toothbrush monofilaments releasing silver ions have recently been developed^21^. However, toothbrushes containing silver ions were reported to have no clear antimicrobial effects against oral bacteria including *S. mutans*^8^. The silver was mainly coated on the surface of the monofilaments^8,21^, and the period of release of silver ions may be transient. In contrast, monofilaments containing S-PRG filler were studded with S-PRG filler buried in the monofilaments in order to allow the ions to release for a longer duration. In fact, the ions were continuously released from the monofilaments and the bacterial inhibitory effect of toothbrushes made with these monofilaments may be long-lasting.

The maximum concentration of S-PRG filler that can maintain the mechanical strength of the monofilaments and continuously spun in a uniform state was 20% for nylon and 1.4% for polyester. Because of these differences in the amount of S-PRG filler, the concentration of ions released from the nylon monofilaments was higher than the concentration of ions released from the polyester monofilaments. This result is consistent with previous papers, in which the amount of ions is correlated with the concentration of S-PRG filler^23^. BO_3_^3−^ and F^−^, major ions that have an inhibitory effect on *S. mutans*^23^, were released from both the nylon and polyester monofilaments containing S-PRG filler, which may add to the inhibitory effect on oral bacteria of these monofilaments.

Tensile strength tests showed that the nylon monofilaments containing S-PRG filler had a lower tensile strength than nylon monofilaments without S-PRG filler. This may be due to the addition of 20% S-PRG filler. Additionally, the tensile strength was lower in the nylon monofilaments immersed in water, regardless of the presence or absence of S-PRG filler, which is consistent with a previous study^24^. However, nylon monofilaments containing S-PRG filler have enough tensile strength to be used as a toothbrush, since the tensile strength of the nylon monofilaments was more than 250 MPa, which is almost the same as polyester monofilaments without S-PRG filler that are commercially available.

One of the major cariogenic properties of *S. mutans* is strong adhesion to the tooth surface in the presence of sucrose^25^. An in vitro experiment to evaluate sucrose-dependent biofilm formation by *S. mutans* has been reported^26^. In the present study, we evaluated the biofilm formation of *S. mutans* on monofilaments in the presence of sucrose with modification to the biofilm assay. *S. mutans* formed biofilm on both the nylon and polyester monofilaments without S-PRG filler, but the amount of biofilm was much less in nylon and polyester monofilaments with S-PRG filler. These results indicate that S-PRG filler dispersed in the monofilaments inhibits biofilm formation on the surface of the monofilament. Nylon monofilaments had a greater inhibitory effect on biofilm formation than polyester monofilaments, possibly because the inhibitory effect on biofilm formation was dependent on the concentration of S-PRG^19^.

A sucrose-dependent adhesion experiment has been developed as an in vitro experiment for evaluating the adhesive properties of *S. mutans* to tooth surfaces in the presence of sucrose^25^. In this experiment, *S. mutans* adheres to the glass surface of a test tube in the presence of a sucrose-supplemented bacterial broth, and the amount of *S. mutans* remaining on the glass surface after vibration is measured. In the present study, we modified the method to evaluate the adhesion of *S. mutans* to the monofilaments instead of the test tube. We found that approximately 40–55% of *S. mutans* remained on monofilaments without S-PRG filler after applying vibration, whereas few *S. mutans* adhered to monofilaments containing S-PRG filler. This finding suggests that *S. mutans* adhering to monofilaments containing S-PRG filler are easily exfoliated.

Many bacteria adhere to toothbrushes after use in daily life^8^. Therefore, chronological changes in the number of *S. mutans* on the monofilaments were evaluated after the monofilament was removed from the bacterial broth. There was a wide difference in the number of *S. mutans* between the monofilaments with S-PRG filler and those without S-PRG filler. Most viable *S. mutans* had disappeared from the monofilaments with S-PRG filler 240 min after the start of the experiment, indicating that toothbrushes containing S-PRG filler harbour fewer viable *S. mutans* after use than conventional toothbrushes.

Nylon monofilaments containing S-PRG filler were shown to delay the bacterial growth of *S. mutans*. Such an inhibitory effect on *S. mutans* in in vitro analysis could translate into inhibition of dental plaque accumulation on the tooth surface and a reduction in the number of *S. mutans* in oral specimens in humans^27^. Therefore, the use of a toothbrush made of these filaments may have an inhibitory effect on *S. mutans* in the oral cavity. However, polyester monofilaments containing S-PRG filler did not affect bacterial growth, possibly because they contain a smaller amount of S-PRG filler-derived ions than do the nylon monofilaments. Recently developed toothpastes containing S-PRG fillers have a remineralizing effect on enamel^28^. If the inhibitory effects of such a toothpaste on *S. mutans* can be confirmed, the combined use of a toothbrush made of monofilaments containing S-PRG filler and this toothpaste may compensate the low inhibitory effect of polyester monofilaments on *S. mutans*.

Future experiments should firstly analyse whether monofilaments containing S-PRG filler have an inhibitory effect on oral bacteria other than *S. mutans,* such as periodontopathic bacterial species, since periodontal disease is not only associated with tooth loss but also with systemic disease^29^. Additionally, clinical trials targeting human subjects should be performed using toothbrushes with monofilaments containing S-PRG filler. The clinical experiments should measure the amounts of oral bacteria adhering to the toothbrushes and evaluate how effectively these toothbrushes clean the tooth surfaces.

In summary, S-PRG filler dispersed within the nylon and polyester monofilaments can release multiple ions. The monofilaments containing S-PRG filler prevented the formation of *S. mutans* biofilm, and the *S. mutans* adhering to the monofilaments were easily exfoliated and eliminated. These results suggest that toothbrushes with monofilaments containing S-PRG filler are effective tools for improving oral health through their inhibitory effect against *S. mutans*.

## Methods

### Analysis of the internal structure of monofilaments containing S-PRG filler

After blending and melting S-PRG filler (Average particle size; 1.0 μm) and resin pellet (nylon 6 or polytrimethylene terephthalate), two types of monofilament containing S-PRG filler (diameter: 200 μm) were spun, respectively. Finally, a nylon monofilament containing 20% of S-PRG filler and a polyester monofilament containing 1.4% of S-PRG filler were respectively produced. At the same time, monofilaments containing no S-PRG filler used as controls were also spun. Each monofilament were cut into a length of 10 cm or 3 cm and used for following analyses. To observe the distribution of S-PRG filler in these monofilaments, SEM images of the transverse section and side structure of each monofilament were taken using G2 Pro (JASCO International Co., Tokyo, Japan). Additionally, elemental analysis of particulate materials present in the monofilaments containing S-PRG filler was performed using energy dispersive X-ray spectroscopy analysis with the G2 Pro, according to the manufacturer's instructions.

### Ion concentration released from monofilaments containing S-PRG filler

First, 360 pieces of monofilaments with a length of 10 cm were immersed in 15 ml sterilised distilled water and rotated at 100 rpm for 18 h. The amounts of the six ions (BO_3_^3−^, Al^3+^, SiO_3_^2−^, Sr^2+^, Na^+^ and F^−^) released from the monofilaments into the water were measured. In addition, six ions not derived from S-PRG filler (Pt^2+^, Ag^+^, Zn^2+^, Cu^2+^, Ca^2+^ and Mg^2+^) were measured. The concentrations of ions released from the S-PRG filler except for F^−^ were measured using Inductively Coupled Plasma Atomic Emission Spectroscopy (ICPS-8100, Shimadzu Co., Kyoto, Japan), as described previously^19^. The concentration of F^−^ was confirmed with a F^−^ Electrode (Model 9609BNWP, Orion Research Inc., Beverly, MA, USA) using an Ion Selective Electrode Meter (Model 720A, Orion Research Inc.), as described previously^19^. In order to examine the sustained release of ions from the monofilament containing S-PRG filler, the measurement of ion concentration was performed at different time points (1, 3, 5, 7, 24, 48, 72, 96, and 168 h after immersion in water).

### Tension test using monofilaments containing S-PRG filler

A tension test of monofilaments with a length of 3 cm before and after immersion in sterilised water was performed using a 5967 Universal Testing Machine (Instron, Norwood, MA, USA). The tensile speed was 10 mm/min, and the tensile strength was measured.

### Inhibitory effects of monofilaments containing S-PRG filler on bacterial mass formation of *S. mutans*

*S. mutans* strain MT8148 (serotype *c*)^30^ was added to 1 ml of BHI broth containing 1% sucrose in a sterilised microtube to a final concentration of 1.0 × 10^7^ CFU/ml. At the same time, each 3 cm monofilament was put into the bacterial broth, which was incubated at 37 °C for 18 h to allow *S. mutans* to adhere to the monofilament. The monofilament was then gently transferred from the broth to sterilised phosphate buffered saline (PBS), and the bacteria were removed from the monofilament by vortexing at 3,000 rpm for 10 s and sonicating at a frequency of 28 kHz for 10 s. The PBS containing *S. mutans* was serially diluted and cultured on Mitis Salivarius Agar (Difco Laboratories) containing Bacitracin (0.2 U/ml; Sigma Chemical Co., St. Louis, MO, USA) and 15% (wt/vol) sucrose (MSB agar). After incubation at 37 °C for 48 h, the number of colonies on the agar plates was counted to determine the number of *S. mutans* present in the monofilament. In addition, the *S. mutans* adhering to the monofilament were observed using a stereoscope and confocal laser microscope. To confirm that the *S. mutans* had successfully exfoliated from the monofilament, the monofilament was put into sterilised PBS and vortexed and sonicated for 1 min. The *S. mutans* cells were mechanically removed from the monofilament with a sterilised chip, and the bacterial suspension was streaked onto an MSB agar plate to count the number of colonies. We confirmed that more than 99.9% of *S. mutans* were exfoliated from the monofilament.

### Inhibitory effects of monofilaments containing S-PRG filler on the adhesive properties of *S. mutans*

*S. mutans* MT8148 was added to 1 ml of BHI broth containing 1% sucrose in a sterilised microtube to a final concentration of 1.0 × 10^7^ CFU/ml. At the same time, each 3 cm monofilament was placed in the bacterial broth, which was incubated at 37 °C for 18 h to allow *S. mutans* to adhere to the monofilament. The monofilament was then gently transferred from the broth to sterilised PBS, and the microtubes were vigorously vibrated with a vortex mixer for 3 s. Then, the solution containing the bacteria detached by vortexing was serially diluted and cultured on MSB plates. Additionally, the monofilament with the remaining adherent bacteria was transferred to another microtube containing PBS, which was vortexed and sonicated to remove all bacteria. The bacterial suspensions were seeded in MSB plates and cultured at 37 °C for 48 h. The rate of adherent *S. mutans* was calculated as 100 × (number of adherent bacteria)/{(number of detached bacteria) + (number of adherent bacteria)}. Additionally, the monofilament was observed with a stereoscope and confocal laser microscope after detachment of *S. mutans* by the first 3 s of vibration.

### Observation of bacterial mass with a confocal laser microscope

The bacterial biofilm formed on the monofilament was analysed using confocal laser scanning microscopy as described previously^31^, with some modifications. The *S. mutans* MT8148 adhering to the monofilaments were resuspended in 1 ml of sterile distilled water including 5 µl of 10 mM Hexidium Iodide (Invitrogen, Carlsbad, CA, USA) and incubated in the dark for 15 min at room temperature. The monofilament was washed with Hanks’ Balanced Salt Solution (Lonza, Walkersville, MD, USA), fixed with 4% paraformaldehyde, and mounted on a slide glass. The bacterial mass of *S. mutans* on the monofilament was observed by confocal scanning laser microscopy using a TCS-SP5 Microscope (Leica Microsystems GmbH, Wetzlar, Germany) with reflected laser light at 543 nm, as well as a DMI6000 B Fluorescence Microscope (Leica) and a 63 × oil immersion objective.

### Chronological changes in the number of *S. mutans* on S-PRG filler-containing monofilaments

After *S. mutans* adhered to the monofilament as described above, the monofilament was removed from the broth and left at room temperature under dry conditions. After 15, 30, 60, 120 and 240 min, the monofilament was put into a microtube containing PBS, followed by vortexing and sonication to remove *S. mutans*. The bacterial suspensions were then seeded on MSB plates and cultured at 37 °C for 48 h, and the number of bacterial colonies was counted.

### Inhibitory effects of monofilaments containing S-PRG filler on bacterial growth of *S. mutans*

Growth assays were performed according to methods described previously with some modifications^19^. *S. mutans* MT8148 was added to 1 ml of BHI broth in a sterilised microtube to a final concentration of 1.0 × 10^7^ CFU/ml. At the same time, 100 pieces of each monofilament with a length of 3 cm were put into the bacterial broth. After incubation at 37 °C for 3, 6, 9, 12 and 24 h, bacterial suspensions were streaked onto MSB plates and incubated anaerobically at 37 °C for 48 h, and the numbers of colonies were counted.

### Statistical analysis

Statistical analyses were conducted using GraphPad Prism 6 (GraphPad Software Inc., La Jolla, CA, USA). Comparisons between two groups were performed using a chi-square test. Intergroup differences in each analysis were determined using analysis of variance (ANOVA). Bonferroni correction was used for post hoc analysis. Results were considered to be significantly different at *P* < 0.05.

## Supplementary Information

Supplementary Information.
