# Supplementary material for: Inhibitory effect of toothbrush monofilament containing surface pre-reacted glass-ionomer (S-PRG) filler on Streptococcus mutans
Source: Sci Rep. 2021 Jan 8;11:211. doi: 10.1038/s41598-020-80646-x (PMC7794465; doi:10.1038/s41598-020-80646-x)
Supplement: Supplementary file 1 — Supplementary Information. [file 41598_2020_80646_MOESM1_ESM.pdf]

Inhibitory effect of toothbrush monofilament containing surface pre-reacted glass-ionomer (S-PRG) filler on *Streptococcus mutans*

Saaya Matayoshi, Ryota Nomura, Takahiro Kitamura, Rena Okawa, and Kazuhiko Nakano

Supplementary Table 1. Weight concentration of elements of the particulate material in nylon filaments analysed by energy dispersive X-ray spectroscopy

Element	Weight concentration (%)
Borate	28.7
Aluminium	12.8
Silicate	12.9
Strontium	40.2
Sodium	1.0
Fluoride	4.4

Supplementary Table 2. Weight concentration of elements of the particulate material in polyester filaments analysed by energy dispersive X-ray spectroscopy

Element	Weight concentration (%)
Borate	16.6
Aluminium	13.0
Silicate	17.9
Strontium	51.2
Sodium	1.3
Fluoride	0.0

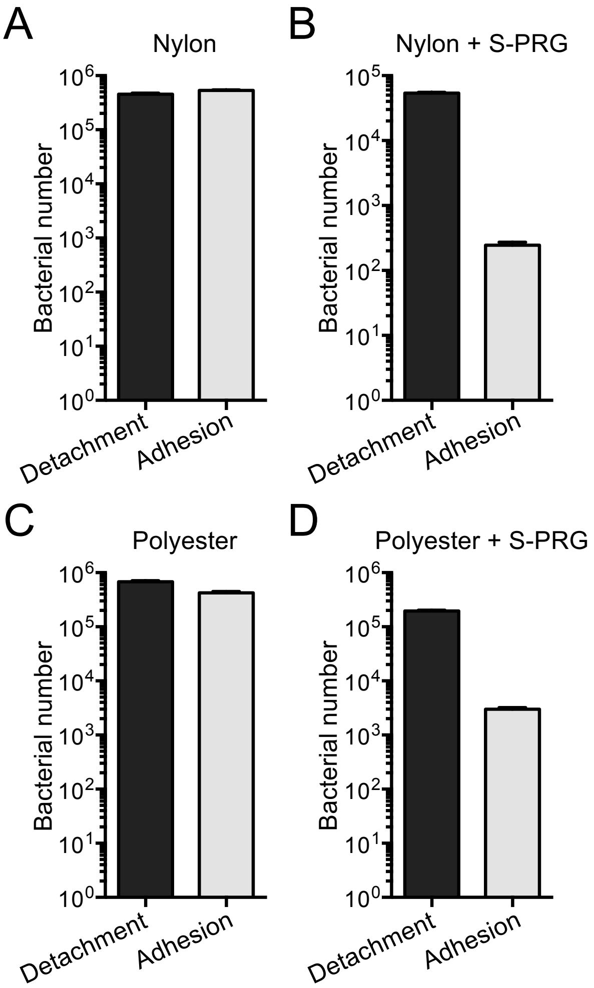

Supplementary Figure 1 Bacterial number of *S. mutans* detached from or attached to the bristle filaments. Nylon filament (A), nylon filament containing S-PRG filler (B), polyester filament (C) and polyester filament containing S-PRG filler.
